# Insanity in Horses

**Published:** 1875-07

**Authors:** 


INSANITY IN HORSES.—We wish some of our California readers would take the trouble to gather and send to us some of the seed-pods of what is known there as the "rattle-weed." We have seen it in quantities, in Monterey county, and further south. The plant grows to the height of about eighteen inches. Its pods are very numerous, and are of a light-brown color when ripe,and though small, are three-sided like the Brazil nut.

Horses eat these nuts and become crazy. They forget to eat or drink, and wander about as if in a maze. However wild they may have been, they are rendered so tame that they can readily be handled. They will walk over a precipice as readily as on safe ground; and seem to lose, under the influence of the plant, what wits they originally posessed.

When thus affected, many of them die; but whether they die from starvation or poison, is not known. If a quantity of the pods is mailed to us, we will subject it to tests which will determine its nature, and will publish the result for the benefit of the world.

---

Make no more vows to perform this or that. It shows no great strength, and makes thee ride behind thyself.

THE VAN DEREN REMEDY—The demands on us for packages of this remedy for summer-diseases of children, under the system of donation announced last month by Hall & Ruckel, come thick and fast.

All who apply will be supplied freely, and we hope and beleive that much good will result from the distribution.

---

A WIDELY-KNOWN INSTITUTION—It was pleasant and curious to notice, during our late trip to the mountains, how wide spread is the knowledge of our Journal and its work. Wherever we went it was sufficient for us to intimate that we were a laborer in the great field which has been cultivated for so many years by HALL'S JOURNAL OF HEALTH. Strangers would step up to us and say, with extended hand, "I am a subscriber to your journal and it has done me *so much* good!" Nearly everybody was ready to declare either that they were subscribers or that they had been, and meant to be again. This recognition was most gratifying.

---

VEGETABLES should never be washed till they are ready to use. Lettuce is made almost worthless in flavor, by dipping it in water a few hours before it is served. Potatoes suffer even more than other vegetables, from the washing-process, and should not touch water till the minute before they are put to cook.

---

EAT rapidly and you will digest slowly.

